# Arthroscopic Pan-Capsular and Transverse Humeral Ligament Release with Biceps Tenodesis for Patients with Refractory Frozen Shoulder

**DOI:** 10.3390/medicina58121712

**Published:** 2022-11-23

**Authors:** Chih-Hao Chiu, Huan Sheu, Poyu Chen, Dan Berco, Yi-Sheng Chan, Alvin Chao-Yu Chen

**Affiliations:** 1 Department of Orthopedic Surgery, Linkou Chang Gung Memorial Hospital, Taoyuan 333, Taiwan; 2 Bone and Joint Research Center, Linkou Chang Gung Memorial Hospital, Taoyuan 333, Taiwan; 3 Comprehensive Sports Medicine Center (CSMC), Linkou Chang Gung Memorial Hospital, Taoyuan 333, Taiwan; 4 Department of Orthopedic Surgery, Taoyuan Chang Gung Memorial Hospital, Taoyuan 333, Taiwan; 5 Department of Occupational Therapy, Graduate Institute of Behavioral Sciences, College of Medicine, Chang Gung University, Taoyuan 333, Taiwan; 6 Healthy Aging Research Center, Chang Gung University, Taoyuan 333, Taiwan; 7 Department of Electronics Engineering and Program in Nano-Electronic Engineering and Design, Chang Gung University, Taoyuan 333, Taiwan; 8 Department of Orthopedic Surgery, Keelung Chang Gung Memorial Hospital, Keelung 204, Taiwan

**Keywords:** refractory frozen shoulder, capsular release, transverse humeral ligament, biceps tenodesis

## Abstract

Arthroscopic capsular release allows direct visualization and release of inflamed tissues in refractory frozen shoulder. The reticular neural network in the long head of the biceps tendon (LHBT) and nerve endings of the transverse humeral ligament (THL) might be responsible for shoulder pain. We hypothesized that patients with painful refractory frozen shoulder benefited from pan-capsular release, THL release, and LHBT tenodesis. The LHBT tenodesis decreased the possibility of LHBT instability. The balance of the shoulder joint was maintained after such extensive release. From October 2013 to June 2019, patients with painful refractory frozen shoulder were enrolled consecutively at the same institute. All patients received arthroscopic pan-capsular, THL release, and suprapectoral LHBT tenodesis with a minimum of 2-year follow-up. Preoperative and postoperative shoulder range of motion (ROM), pain visual analog scale (PVAS), subjective shoulder value (SSV), constant score, LHBT score, acromio-humeral distance (AHD), and critical shoulder angle (CSA) were recorded. In total, 35 patients with an average age of 53.1 ± 9 years were enrolled. The average follow-up period was 24 ± 1.5 months. Forward elevation improved from 105.1° ± 17° to 147° ± 12° (*p* < 0.001), external rotation improved from 24.1° ± 13.3° to 50.9° ± 9.7° (*p* < 0.001), and internal rotation improved from L3 to T9 (*p* < 0.001), respectively, at final follow-up. PVAS improved from 7.3 ± 1.1 to 1.8 ± 0.6 (*p* < 0.001), constant score from 23.4 ± 11 to 80.7 ± 5.2 (*p* < 0.001), and SSV from 27.7 ± 10.5 to 77.4 ± 3.8, respectively, at follow-up. No differences were found in AHD and CSA after surgery (*p* = 0.316, and *p* = 0.895, respectively). Patients with painful refractory frozen shoulder benefited from pan-capsular and THL release. A radiographically balanced shoulder joint was maintained even after such extensive release.

## 1. Introduction

Frozen shoulder is an inflammatory disease with spontaneous onset impairing glenohumeral motion [1], and it is often regarded as a self-limiting disease that resolves within a range of 1 to 3 years. However, 20 to 50% of patients have been reported to still have long-lasting symptoms in various studies [2,3]. First-line treatments for frozen shoulder include analgesia, physiotherapy [4], intra-articular corticosteroid injection [5], hydrodilation [6], and manipulation under anesthesia. Arthroscopic capsular release, although safe and effective, is only reserved for shoulders refractory to conservative treatment measures [7]. When compared to manipulation under anesthesia and hydrodilation, arthroscopic capsular release allows for the direct visualization and release of the tightened coracohumeral ligament (CHL), thickened rotator interval (RI), and contracted capsule to ensure adequate release [8]. Patients who received arthroscopic capsular release showed a significantly higher Oxford shoulder score at 6 months than those receiving hydrodilation [9]. Several other groups reported quick and long-lasting pain relief effects after arthroscopic capsular release [7,10].

However, there is still controversy regarding the extent of release in refractory frozen shoulder. Ogilvie-Harris et al. advocated the inferior posterior capsular release in resistant frozen shoulder [11]. Chen et al. concluded that the extended release of the posterior inferior glenohumeral ligament (IGHL), along with anterior capsular structures, improved range of motion (ROM) more rapidly within the first 3 months postoperatively [12]. Hagiwara et al. published their surgical technique of arthroscopic CHL release and considered this procedure essential and reliable for regaining full ROM for frozen shoulder [13]. On the other hand, frozen shoulder often presents with diffuse shoulder pain followed by gradual loss of both active and passive ROM, which made it difficult to evaluate the bicipital groove area because of the close anatomical proximity of the structures in the anterior shoulder [14]. The chronic inflammation of synovium causes mechanical impingements in capsular tissue around the bicipital groove [15], and adhesions within a tenosynovial compartment of the long head of the biceps tendon (LHBT). LHBT also had a reticular neural network containing sensory and sympathetic neurotransmitters presented around the tendon. Not only the nociception, but the neurotransmitters within had pivot roles in the vasoregulation and immunomodulation of neurogenic inflammation [16]. Additionally, transverse humeral ligaments (THL) demonstrated myelinated and unmyelinated free nerve endings, suggesting a potential role as a pain generator of anterior shoulder pain [17].

There is a paucity of literature evaluating the role of capsular and THL release and LHBT treatment in refractory frozen shoulder. The purpose of this study is to report our result of arthroscopic pan-capsular and THL release with LHBT tenodesis for patients with refractory frozen shoulder. We hypothesized that patients with a painful refractory frozen shoulder could be benefited from pan-capsular release, THL release, and LHBT tenodesis. The associated LHB tenodesis could decrease the possibility of LHBT instability. The balance of the shoulder joint could be maintained even after such extensive release and LHBT tenodesis.

## 2. Materials and Methods

### 2.1. Patient Enrollment

From October 2013 to June 2019, patients with refractory frozen shoulder were enrolled in this retrospective study. The written informed consent was obtained from all participants enrolled in this study. The inclusion criteria of refractory frozen shoulder involved moderate-to-severe shoulder pain [18] around the bicipital groove with no improvement after conservative treatments, such as local steroid injections or physiotherapies, for at least six months, and limited ROM of the shoulder with anterior elevation being up to 130°, external rotation up to 50°, and internal rotation up to L5 [19]. The exclusion criteria included radiological signs of fracture, glenohumeral arthritis, previous LHBT tenodesis/tenotomy, and concomitant rotator cuff tear with more than 50% of footprint involvement. All enrolled patients received arthroscopic pan-capsular and THL release with suprapectoral LHBT tenodesis at the entry of the bicipital groove (top-of-the-groove tenodesis) with a double-loaded anchor. The authors explained the methods to all participants enrolled in this study. All surgeries were performed by a single surgeon in the same institute. This study was approved by the Institutional Review Board of the author’s institutes (IRB 201900352B0).

### 2.2. Outcome Assessment

Preoperative demographic data included sex; age; involvement of the dominant arm; duration of pain; number of previous injections; and underlying diseases, such as diabetes, breast cancer, thyroid dysfunction, cancer, and previous traumatic history. Degrees of passive maximum forward elevation and external rotation were measured by a goniometer. Internal rotation was measured by the vertebral spinous process that could be reached with the tip of the patient’s thumb and was converted into contiguously numbered groups: T1-12 to 1-12, L1-5 to 13–17, buttock to 18, and greater tubercle of the proximal femur to 19 [20]. All patients had preoperative X-ray, ultrasonography exam, and magnetic resonance imaging (MRI) to evaluate the conditions of the rotator cuff, labrum, capsules, and LHBT. The ultrasonography was performed in consensus by two independent observers: one musculoskeletal radiologist and one orthopedic surgeon different from the operating surgeon. All the ultrasonography scans were performed using a Medison ACCUVIX V20 machine with 7.5–13 MHz linear array transducer (Medison America, Inc., Cypress, CA, USA). Standard scanning techniques and positions were used as described in previous studies [21,22]. The OMERACT US definitions for tenosynovitis, synovitis, synovial hypertrophy, and effusion were applied [23]. The criteria for biceps tendinosis were defined as the presence of an increased Doppler signal, plus one of the following features: (1) thickening of the biceps tendon; (2) evidence of synovial thickening of more than 3 mm; (3) evidence of fluid collection in the biceps tendon sheath of more than 3 mm; and (4) splitting and hypoechoic change in the biceps tendon or a negative Doppler signal but with two of the above mentioned features, as previously described [24]. Constant score [25], pain visual analog scale (PVAS), subjective shoulder value (SSV) [26], ROM, O’Brien test, speed tests, and tenderness over the LHBT were investigated before the operation and at final follow-up.

For radiological evaluation, the patients were evaluated preoperatively using a standardized radiographic examination (a true anteroposterior (AP) radiograph with the arm in neutral rotation). The acromio-humeral distance (AHD) was measured in millimeters on the AP radiograph. The critical shoulder angle (CSA) was measured between a line connecting the inferior with the superior border of the glenoid fossa and another connecting the inferior border of the glenoid with the most inferolateral point of the acromion [27]. Two independent assessors assessed the AHD and CSA preoperatively and 2-year postoperatively.

The ultrasound scan for the LHBT and rotator cuff integrity was performed preoperatively and at final follow-up. LHBT were obtained in longitudinal and axial planes with the forearm in supination and the elbow in 90° of flexion. The anatomic failure of tenodesis was determined when the LHBT fibers were not traced longitudinally in the intertubercular groove [28]. A finding of a rotator cuff tear was defined when there was a focal defect extending from the bursal to the humeral side of the tendon or complete non-visualization of the tendon [29].

### 2.3. Surgical Technique

All patients were placed in the beach chair position under general anesthesia with interscalene block. ROM of the involved shoulder was examined in light force to rule out patients with a pseudo-frozen shoulder [30]. Clinically, we found some included patients with frozen shoulders revealed decreased ROM during consultation because of muscle guarding and pain misdiagnosed with a frozen shoulder but had better mobility under anesthesia. Manipulation was avoided to decrease the incidence of complications, such as fractures of the humerus [31]. Traction device was not used to facilitate intra-operative shoulder ROM to see if there was residual stiffness after release.

### 2.4. Intra-Articular Release

**Figure 1:**
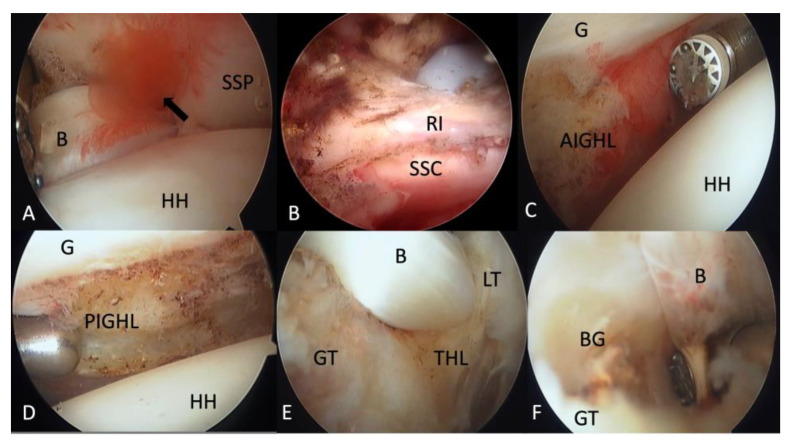
Surgical technique to release right refractory frozen shoulder. (**A**) Viewing from posterior portal of right shoulder. Lots of synovium fluid (arrow) extravasated from the bicipital groove when pulling the LHBT medially with a probe. (**B**) Thorough release of rotator interval with a radiofrequency wand. (**C**) The radiofrequency wand was extended downward around the 6 o’clock position to release the anterior band of the inferior glenohumeral ligament. (**D**) Viewing from anterior portal with the radiofrequency wand introduced from posterior portal. The posterior IGHL should be released until the long head of the triceps was seen. (**E**) Viewing from anterolateral portal in the subacromial space. The greater and lesser tuberosities were used to define the location of the LHBT and transverse humeral ligament. (**F**) Two tissue layers of transverse humeral ligament were all released. The LHBT was pulled medially to reveal the bicipital groove. Any scar, adhesion, abrasive osteophyte, and loose bodies should be removed at this time. AIGHL, anterior band of inferior glenohumeral ligament; B, LHBT; BG, bicipital groove; G, glenoid; GT, greater tuberosity; HH, humeral head; LT, lesser tuberosity; PIGHL, posterior band of inferior glenohumeral ligament; RI, rotator interval; SSC, subscapularis; SSP, supraspinatus; THL, transverse humeral ligament.

The diagnostic arthroscopy with a 30° arthroscope (1488 Full HD; Stryker, San Jose, CA, USA) was firstly introduced from the standard posterior portal. Then, an anterior portal was made with an outside-in technique using a 23-gauge needle. The trajectory of the needle was parallel to the glenoid and the percutaneous entrance was lateral to the coracoid. In this approach, we could make sure the radiofrequency wand reached the bottom of the shoulder joint without damaging cartilages of the humeral head or glenoid. The LHBT and superior labrum were examined by the probe to see if there was instability or tear. Lots of synovium fluid was often found extravasate from the bicipital groove when pulling the LHBT medially with a probe (Figure 1A). The CHL and superior glenohumeral ligament (SGHL) were also cut to make sure there was no proximal impingement of the LHBT when exiting the groove. If there was adhesion between the LHBT and the undersurface of the supraspinatus, the release was performed until the LHBT could slide freely. The rotator interval was always covered by thick and erythematous synovium and fibrous tissues, as well as neovascularization, in refractory frozen shoulder. Thorough release of RI (Figure 1B) was performed with the radiofrequency wand until there was no scar tissue between the subscapularis and coracoid. The medial glenoid humeral ligament (MGHL) was cut when impingement was found during subscapularis excursion. The radiofrequency wand was then extended downward around the 6 o’clock position to release the anterior band of the IGHL until the muscle part of the subscapularis was revealed (Figure 1C). Then, the arthroscopy was shifted to the anterior portal and the radiofrequency wand from the posterior portal. The shoulder was manually kept in 45 degrees abduction and neutral rotation because the axillary nerve moved farther away than in the neutral arthroscopic setup position [32]. The release of the middle and posterior band of IGHL was performed as close as possible to the glenoid rim because the axillary nerve is closer to the humeral insertion of the capsule [33]. Posteriorly, the release should involve the most superior band of the posterior IGHL until the long head of the triceps tendon was confirmed (Figure 1D). If partial articular supraspinatus tendon avulsion (PASTA) <50% of footprint involvement was found during the scope exam, it would be debrided and left alone without repair.

### 2.5. THL Release

After intra-articular release, the scope was shifted to subacromial space viewing from the lateral portal. An anterolateral portal was also made to facilitate subacromial release. Coracoacromial ligament (CAL) was preserved and acromioplasty was not performed. After debridement of subacromial bursitis, the shoulder was put in an abduction–external rotation position to see the anterior portion of the extra-articular shoulder joint. The LHBT was easily located by probing the bicipital groove or pulling medially out of the groove through the anterior portal. Greater and lesser tuberosity could be used to define the location of LHBT (Figure 1E). Viewing from the anterolateral portal, THL was released with a radiofrequency wand introduced from the anterior portal. The lateral border of the opened rotator interval provided a good reference to start the release distally along with the anatomy of the LHBT. Two tissue layers of the THL were all released. Then, the LHBT was pulled medially to reveal the bicipital groove (Figure 1F). Any scar, adhesion, abrasive osteophyte, and loose bodies should be removed at this time.

Following the release, a double-loaded suture anchor was inserted at the junction of the bicipital groove and humeral head cartilage. One lasso loop and one simple stitch from the same suture were passed through LHBT and fixed with 7 knots. The other suture was passed through the LHBT in the same way 5 mm proximal to the first knot to secure the LHBT. Finally, the fixed LHBT was cut at the level of the LHBT anchor. Care should be taken not to leave an intra-articular huge stump.

### 2.6. Postoperative Care

All patients had sling protection for the initial 2 weeks right after the surgery. Then, an aggressive rehabilitation program began with passive ROM exercises from 2 to 6 weeks postoperatively. This was followed by active-assisted ROM exercises from 6 to 12 weeks under the supervision of physical therapists in the hospital or self-stretches at home. All patients had follow-up consultations at 2 weeks, 6 weeks, 6 months, and 2 years after surgery. An ultrasonography exam was performed at the final follow-up to see the integrity of the rotator cuff and LHBT by the first author, who had more than 10 years of experience in surgery and ultrasonography.

### 2.7. Statistical Analysis

All statistical analyses were performed with IBM SPSS 25.0 for Mac (SPSS Inc., Armonk, NY, USA). Continuous data were described by means and standard deviations. Paired *t*-tests were used to analyze the difference between pre- and postoperative outcome scores for constant score, PVAS, SSV, ROM, AHD, and CSA, respectively. Two-tailed *p* values of less than 0.05 were considered significant. An a priori power analysis was conducted to find the minimum sample needed to detect a difference in the preoperative and postoperative PVAS, SSV, ROM, AHD, and CSA. With alpha = 0.05, power set at 80%, and a standard deviation of 10, a minimum of thirty-three patients in each group was needed [34].

## 3. Results

**Figure 2:**
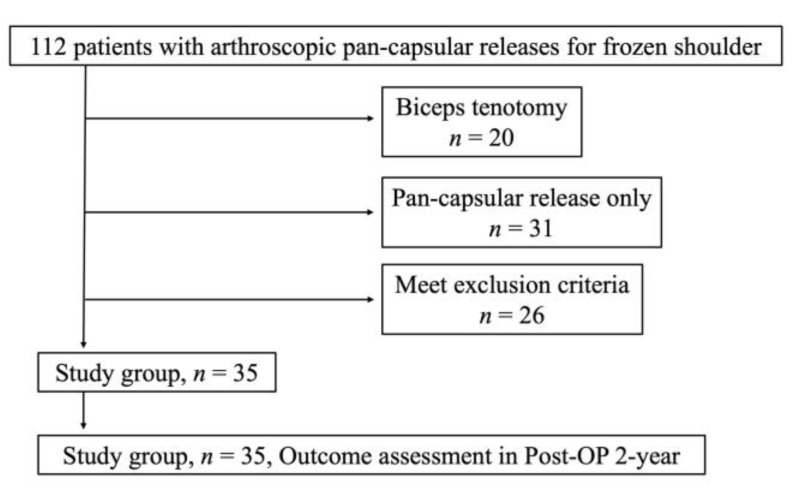
Study flow chart.

The author performed 112 arthroscopic capsular releases for frozen shoulders. Of them, 20 patients were excluded because LHBT tenotomy was performed during the surgery and another 31 patients were excluded due to having only pan-capsule release without LHBT treatment. A total of 26 patients failed to attend the minimum requirement of at least a 2-year follow-up consultation, leaving 35 patients enrolled in the study (Figure 2).

### 3.1. Cohort Demographics

The study included 19 men (54%) and 16 women (46%), with a mean age of 53.1 ± 9 (mean ± standard deviation) years (range, 36–74 years). The mean duration of symptoms was 9.9 ± 2.3 months (range, 6–12 months). Of the 35 operated shoulders, 15 (43%) were on the right side and 20 (57%) were on the left. A total of 30 patients (86%) were right hand dominant, and 5 (14%) had left-hand dominance. The average number of injection (steroid, hyaluronic acid, platelet-rich plasma, etc.) before the index surgery was 5.2 ± 2.1. The patient demographic data were listed in Table 1.

### 3.2. Range of Motion

**Figure 3:**
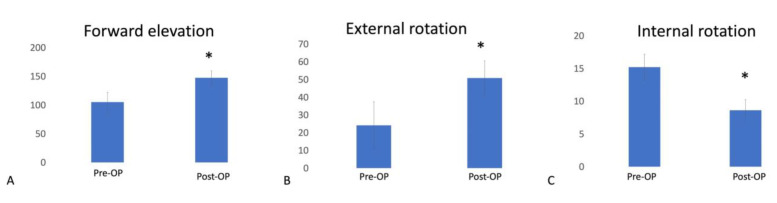
Preoperative and postoperative range of motion. (**A**) Forward elevation improved from 105.1° ± 17° to 147° ± 12°. (**B**) External rotation improved from 24.1° ± 13.3° to 50.9° ± 9.7°. (**C**) Internal rotation improved from 15.2 ± 2 (L3) to 8.6 ± 1.6 (T9). * *p* value < 0.001.

After the surgery, all patients experienced significant improvement in active ROM. Preoperative forward elevation ranged from 105.1° ± 17° (mean ± standard deviation), and this increased to 147° ± 12° (*p* < 0.001) at final follow-up (Figure 3A). External rotation improved from 24.1° ± 13.3° to 50.9° ± 9.7° (*p* < 0.001) (Figure 3B), and internal rotation improved from 15.2 ± 2 (L3) to 8.6 ± 1.6 (T9) (*p* < 0.001) at final follow-up (Figure 3C).

### 3.3. Functional Assessment

**Figure 4:**
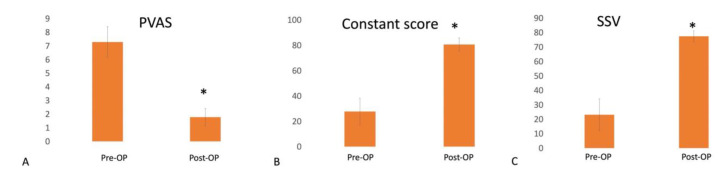
Preoperative and postoperative functional assessment. (**A**) PVAS decreased from 7.3 ± 1.1 to 1.8 ± 0.6. (**B**) Constant score improved from 23.4 ± 11 to 80.7 ± 5.2. (**C**) SSV improved from 27.7 ± 10.5 to 77.4 ± 3.8. PVAS, pain visual analog scale; SSV, subjective shoulder value. * *p* value < 0.001.

Before their operation, all patients had pain during the movement, positive O’Brien test, speed tests, and tenderness over the LHBT. The preoperative PVAS was 7.3 ± 1.1 and improved to 1.8 ± 0.6 (*p* < 0.001) at the final follow-up. The constant score improved from 23.4 ± 11 to 80.7 ± 5.2 (*p* < 0.001), and SSV improved from 27.7 ± 10.5 to 77.4 ± 3.8 (*p* < 0.001), respectively, at final follow-up (Figure 4).

### 3.4. Image Assessment

**Figure 5:**
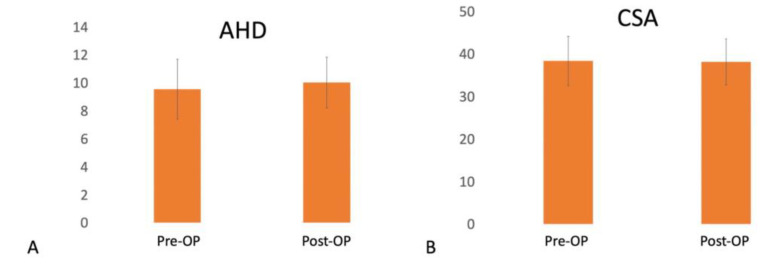
Preoperative and postoperative image assessment. (**A**) Preoperative AHD was 9.5 ± 2.1, and postoperative AHD was 10 ± 1.8, *p* = 0.316. (**B**) Preoperative CSA, 38.3 ± 5.8, and postoperative CSA, 38.2 ± 5.4, *p* = 0.895. AHD, acromio-humeral distance; CSA, critical shoulder angle, Pre-OP, preoperative; Post-OP, postoperative.

All patients had thickened CHL and capsular thickening in the axillary recess on preoperative MRI. Effusion around the LHBT was commonly observed from ultrasonography and MRI before surgery. There were no significant differences between preoperative and postoperative AHD (9.5 ± 2.1 vs. 10 ± 1.8, *p* = 0.316) and CSA (38.3 ± 5.8 vs. 38.2 ± 5.4, *p* = 0.895) (Figure 5). On ultrasound evaluation at final follow-up, one (5.7%) patient had a retear of their LHBT with Popeye deformity; no rotator cuff tear more than 50% was diagnosed. The eight (22.9%) PASTA lesions remained unchanged in sizes under ultrasound examination.

## 4. Discussion

Our study revealed that patients with refractory frozen shoulder and pain benefited from pan-capsular release, THL release, and LHB tenodesis. In addition, the radiological parameters, such as AHD and CSA, did not change after surgery, implying a radiographic balanced shoulder joint was maintained even after such extensive release. Frozen shoulder is an inflammatory disease with spontaneous onset impairing all the directions of glenohumeral motion [1]. It is often regarded as a self-limiting disease that resolves within 3 years. However, various studies have shown inferior outcomes and long-lasting symptoms with conservative treatments [35,36]. When conservative treatment failed, Smith et al. found that 50% and 80% of patients had good pain relief within 1 and 6 weeks of arthroscopic capsular release [7]. Le Lievre et al. observed all patients had improvement in pain frequency and severity, shoulder function, and ROM at a long-term follow-up of 7 years [10]. On the other hand, LHBT is also a common source of pain in the shoulder, and it is often difficult to identify clinically when LHBT pathology coexists with a frozen shoulder. Intra-articularly, Hagiwara et al. reported the presence of superomedial capsule and the LHBT adhesion with synovitis in diabetic patients with decreased ROM [13]. Extra-articularly, Taylor et al. demonstrated that 47% of chronically symptomatic patients had hidden extra-articular LHBT lesions, such as adhesion, LHBT instability, stenosis, abrasive osteophyte, LHBT partial tear, and loose bodies. Among them, 45% of patients with an intra-articular LHBT lesion also had hidden tunnel lesions extra-articularly. High levels of inflammatory cytokines, degenerative mediators, and nociceptive molecules were also observed within the bicipital groove [37], causing pain in the shoulder and warranting release. Soifer et al. identified neurofilaments and peripheral nerves within the LHBT, tendon sheath, and THL in 14 cadaveric shoulders [38]. Snow et al. demonstrated two tissue layers in THL. Neurohistological staining revealed the presence of free nerve endings in both layers, which suggests THL has a potential role as a shoulder pain generator [17]. Therefore, we enrolled patients with painful refractory frozen shoulders who failed conservative treatment and performed not only pan-capsule release intra-articularly but also THL release extra-articularly to avoid inadequate treatment for such complicated situations. A proximal LHBT tenodesis was also performed to prevent LHBT instability after the extensive release, including the whole RI, SGHL, and THL, which provides soft tissue restraint for LHBT stability. In our result, only one (2.8%) patient had retear or dislocation of fixed LHBT at follow-up. A total of 97.2% of patients had healed LHBT inside bicipital groove.

We did not repair the PASTA lesion when it involved less than 50% of the footprint. One reason is that most studies suggest PASTA repair when there is greater than 50% of the tendon footprint and tendon thickness involved [39]. The other reason is, as Ueda et al. demonstrated, shoulder stiffness with severe and global loss of passive ROM is not associated with full-thickness rotator cuff tears, although some patients may have a partial-thickness tear [40]. We choose not to repair the PASTA lesion because it may be irrelevant to the clinical condition of our patient group. Our results proved that the pan-capsular release without PASTA repair did not significantly change the AHD and CSA of the treated shoulder at final follow-up.

In our study, we preferred THL release to relieve the anterior shoulder pain and suprapectoral LHBT tenodesis to prevent LHBT instability because of the extensive release of RI and SGHL. There are plenty of debates about where to fix the LHBT. Sanders et al. reported a higher revision rate for proximal arthroscopic tenodesis than distal (35.7% vs. 2.7%). However, patients in their group of proximal tenodesis had a lower revision rate (2.4%) when the bicipital sheath was released, compared to those not released (13.4%) [14]. When comparing the result of proximal versus distal tenodesis, Lutton et al. demonstrated that 2 of the 5 patients with tenodesis within the upper half of the bicipital groove had persistent pain at the tenodesis site, requiring further debridement surgery, while the other 12 patients with tenodesis in the lower half of the groove or shaft were free of symptoms [41]. Our study echoed their results by releasing the THL to avoid the unwanted bicipital groove pain after the LHBT tenodesis. Additionally, we wanted to prevent the complications of subpectoral LHBT tenodesis, such as brachial plexus injury [42], musculocutaneous nerve palsy [43], and proximal humeral fractures [44]. Since the LHBT was well-fixed by two sutures, we encourage our patients to move aggressively after the surgery. This may contribute to our good results showing significant improvement in all directions of ROM and functional movement.

There were limitations of this study. First, this was a retrospective study and as such had inherent bias. The second limitation was the indication of the surgery. Most patients with frozen shoulder respond well to conservative treatments. Therefore, we only enrolled patients with refractory frozen shoulders who failed plenty of injections and physiotherapy. Additionally, patients with diabetes did not respond as well toward surgical release as patients without diabetes [45]. In such patients, the author prefers LHBT tenotomy to tenodesis. Further study should focus on these patients to see if they would have lesser, equivalent, or improved outcomes with either arthroscopic pan-capsular release alone, or an arthroscopic pan-capsular release with an LHBT tenotomy. Only 14.3% in our patient cohort had diabetes under good medical control and received LHBT tenodesis. Further study should focus on the role of LHB tendonitis in frozen shoulder and how to treat it, along with pan-capsular release as the close anatomical proximity of LHBT at the anterior shoulder, which makes it “difficult to define, difficult to treat and difficult to explain” as Earnest Codman described [46]. Third, we lacked a control group of either no surgery or pan-capsular release without LHBT treatments, which would have allowed us to compare the groups to determine if this would alter the surgeon’s practice decisions.

## 5. Conclusions

Patients with refractory frozen shoulder and pain benefited from pan-capsular and THL release. A balanced shoulder joint was maintained even after such extensive release.

## Figures and Tables

**Table 1:** Patient demographics. Data are presented as mean ± SD; PASTA, partial articular supraspinatus tendon avulsion; SLAP, superior labrum anterior posterior tear.

No. of Patients	35
Age, y	53.1 ± 9
Sex, male: female, n	19:16
Dominant hand, right: left	30:5
Surgical side, right: left	15:20
Duration of pain, month	9.9 ± 2.3
Number of previous injections	5.2 ± 2.1
Mean follow-up, month	24 ± 1.5
Underlined disease (%)	
Diabetes mellitus	5 (14.3)
Cancer	2 (5.7)
Thyroid dysfunction	0
Previous trauma	4 (11.4)
Intra-operative finding other than frozen shoulder (%)	
PASTA lesion	8 (22.9)
Biceps pulley tear	2 (5.7)
SLAP tear	1 (2.8)

## Data Availability

Not applicable.
